# Supplementary material for: Both electronic and vibrational coherences are involved in primary electron transfer in bacterial reaction center
Source: Nat Commun. 2019 Feb 25;10:933. doi: 10.1038/s41467-019-08751-8 (PMC6389996; doi:10.1038/s41467-019-08751-8)
Supplement: Supplementary file 1 — Supplementary information [file 41467_2019_8751_MOESM1_ESM.pdf]

# Both electronic and vibrational coherences are involved in primary electron transfer in bacterial reaction center

Supplementary Information

Ma *et al*

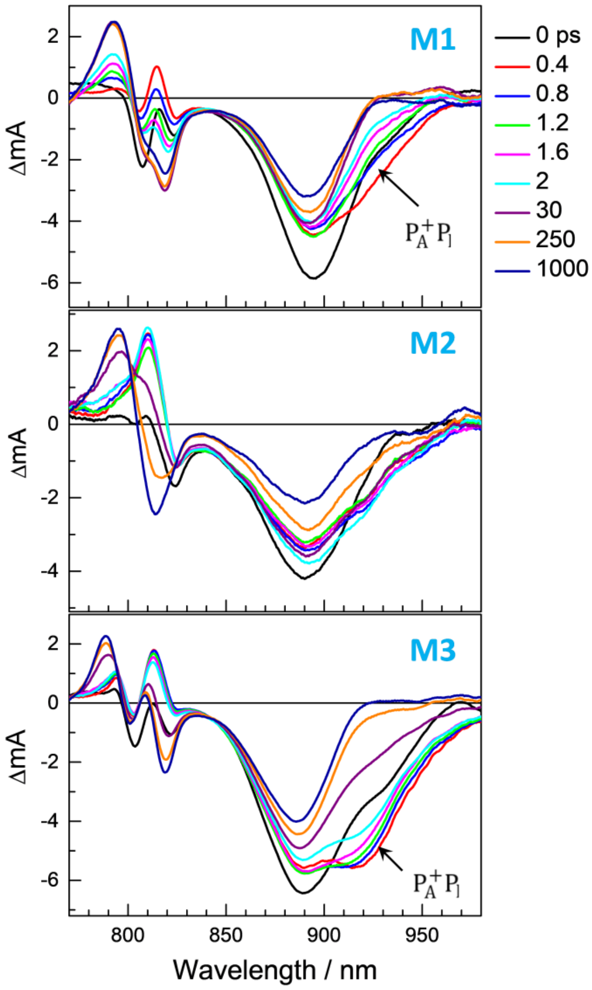

Supplementary Figure 1. 77 K transient absorption spectra of **M1** (top), **M2** (middle) and **M3** (bottom) at selected delay times. The pump and probe pulses were the same with those used in 2DES experiments. The arrows indicate the ground-state bleach signal of $P_A^+P_B^-$.

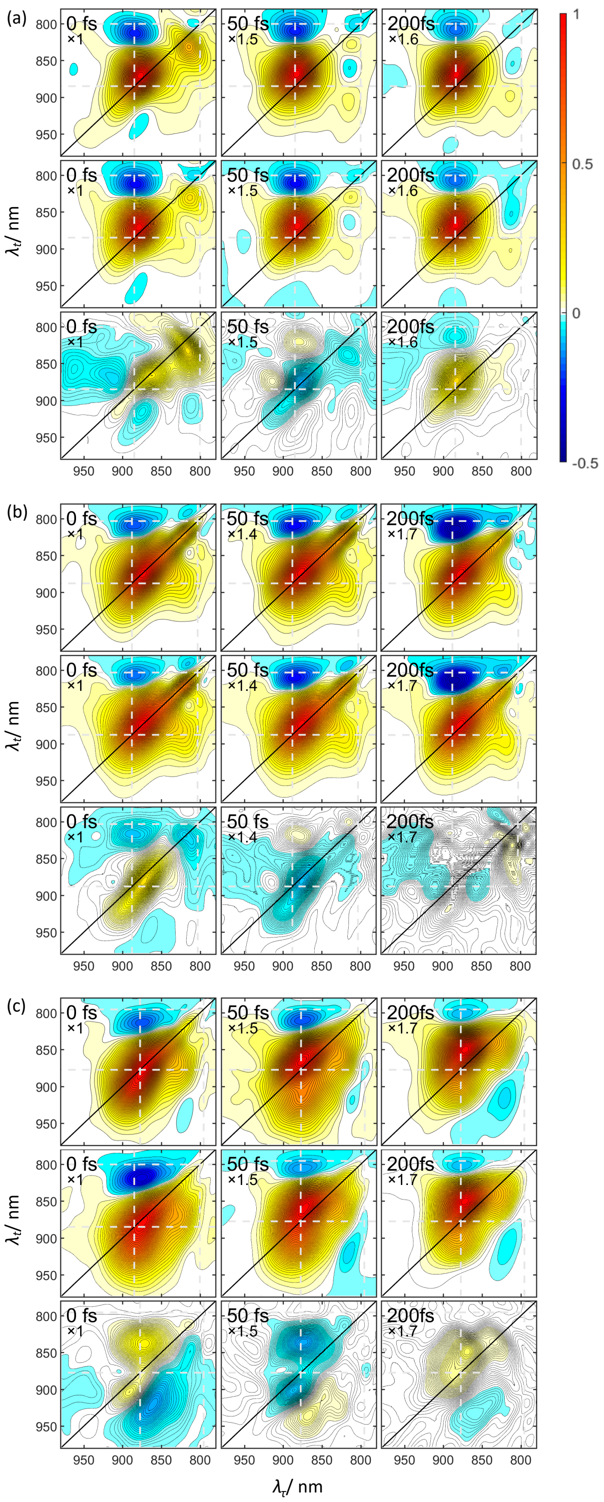

Supplementary Figure 2. Measured 2D spectra (top, same as those in Fig. 2), fitting 2D spectra obtained from global analysis (middle) and residual 2D spectra (bottom) of **M1** (a), **M2** (b) and **M3** (c).

Supplementary Note 1

Unlike M1 and M3, in M2, there were no observable features corresponding to $P_A^+P_B^-$, seen from either the 2DES or the transient absorption spectra. The species evolution dynamics of M2 were fitted well with a three-component model as shown in the main text. Here we show the fitting result with a four-component model and compare them with that of the 3-component model, to confirm the latter's validation.

The 2D-EAS of the four components are shown in Supplementary Figure 3. In Component-2, no clear features corresponding to $P_A^+P_B^-$ appeared. From Component-1, -2 to -3, the main change was the growth of the negative signals, and the spectral shape of Component-2 seemed to be a transitive intermediate of the other two. It indicated that Component-2 and -3 can be attributable to the same species.

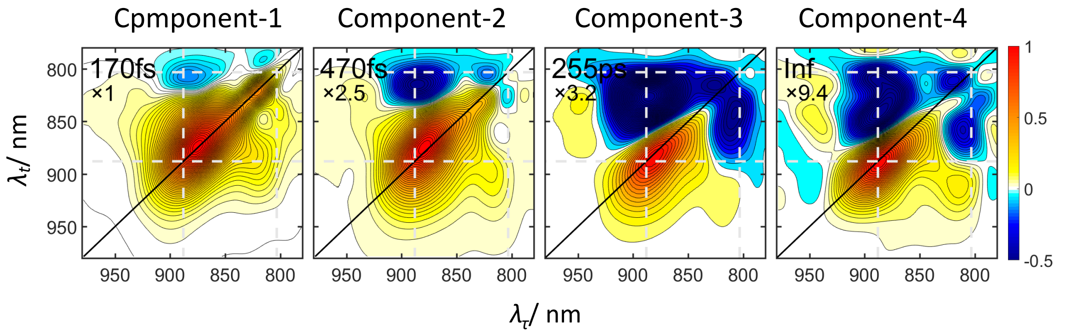

Supplementary Figure 3. 2D-EAS of M2 obtained by fitting with a four-component model. The time constants of each species are shown in the left-top corner of each panel. The spectra are normalized to the maximum of the diagonal P signal; the relative amplitude multiplier is shown below the time constants.

Supplementary Note 2

From 77 K to room temperature (see Supplementary Figure 4), the time constant of $P_A^+P_B^- \rightleftharpoons P^+B_A^-$ increased from 1.4 ps to 3.4 ps. The rise time constants of the positive (B, P) cross peak were identical, 430 fs. However, the amplitude became much more pronounced at room temperature, indicating an increase of electron transfer from B to P. This can be explained by a reversible reaction scheme [1]: when the forward reaction becomes slower, the backward reaction becomes more effective. Hence when the forward electron transfer of $P_A^+P_B^- \rightleftharpoons P^+B_A^-$ became slower, from $(1.4\ \text{ps})^{-1}$ to $(3.4\ \text{ps})^{-1}$, the backward electron transfer, i.e. charge recombination, became more effective.

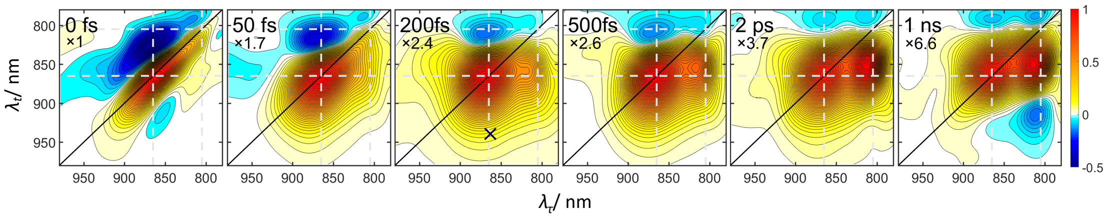

Supplementary Figure 4. Room-temperature absorptive total 2D spectra of **M1.** The indicated population time T is shown in the left-top corner of each panel. The spectra are normalized to the maximum of the diagonal P signal; the relative amplitude multiplier is shown below the T.

Supplementary Note 3

In order to prove that the charge recombination, the backward electron transfer of $P_A^+P_B^- \rightleftharpoons P^+B_A^-$, is responsible for the (B, P) cross peak, we employed kinetic modeling with a reversible reaction. This model did not include a real physical process, but focused on population dynamics and gave a direct view of the dependence of the cross peak amplitude on the reaction rate.

The kinetic model is $C \underset{k_{-1}}{\overset{k_1}{\rightleftharpoons}} D \xrightarrow{k_2}$, with C and D corresponded to $P^*/P_A^+P_B^-$ (because $P^* \rightarrow P^+B_A^-$ is much faster than the succedent process, $P_A^+P_B^- \rightleftharpoons P^+B_A^-$, we treated P* and $P_A^+P_B^-$ as the same component) and $P^+B_A^-$, respectively. The rate equations were:

$$\frac{d[\mathrm{C}]}{dt} = -k_1[\mathrm{C}] + k_{-1}[\mathrm{D}] \tag{1}$$

$$\frac{d[\mathrm{D}]}{dt} = k_1[\mathrm{C}] - k_{-1}[\mathrm{D}] - k_2[\mathrm{D}] \tag{2}$$

The solutions described the population kinetics of C and D. To model the 2DES, four stick spectra corresponding to diagonal and cross peaks were represented with 2D Gaussians, and weighted with the population kinetics. The population kinetics for the CD/DC cross peak were given by the population kinetics of D/C with initial conditions $[C]_0$=1 and $[D]_0$=0/$[D]_0$=1 and $[C]_0$=0.

The 2DES for population time T=10 ps are shown in Supplementary Figure 5, with fixed k_1 and k_2 as $(1\ \mathrm{ps})^{-1}$ and $(0.4\ \mathrm{ps})^{-1}$ and changing k_{-1}. It is clear that the amplitude of the DC cross peak increased with the increasing k_{-1}. The situation of (a)/(b) well represented that of **M1**/**M3**, proving our argument.

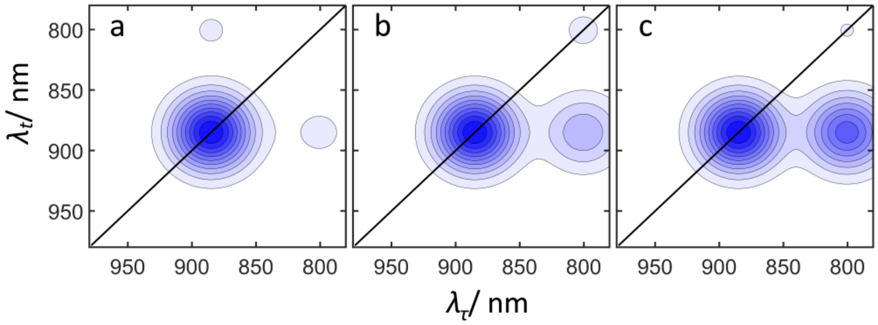

Supplementary Figure 5. 2D spectra of the model system with k_{-1} as (5 ps)$^{-1}$ (a), (1 ps)$^{-1}$ (b) and (0.2 ps)$^{-1}$ (c).

Supplementary Note 4

In **M1**, the rapidly-dephasing (<200 fs) QBs had maximal amplitude around the (P*, $P_A^+P_B^-$) position, which can be seen from the 2D distribution of the amplitudes of the QBs (Supplementary Figure 6). The approximate amplitudes of the QBs were obtained by fitting the 0−200 fs population kinetic traces (after subtraction of the multi-exponential dynamics) using a sine function with a fixed frequency of 195 cm^{-1}. The maximal-amplitude position, (885, 902) nm, is close to the one determined by global analysis, (880, 910) nm. In **M3**, the maximal amplitude position was at (874, 925) nm, slightly shifted from the one determined by global analysis. In **M2**, however, there was nearly no amplitude at (P*, $P_A^+P_B^-$) location. The complex amplitude may reflect the long-lived QBs.

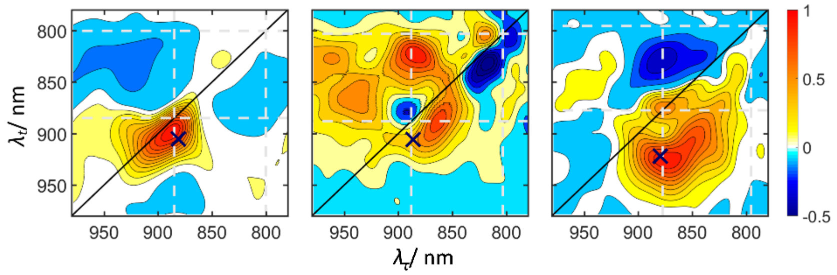

Supplementary Figure 6. 2D amplitude distributions of the short-lived QBs. The negative signals represent the QB amplitudes but with opposite phase. The crosses mark the (P*, $P_A^+P_B^-$) cross-peak location as shown in Fig. 3.

Supplementary Note 5

At room temperature, the QBs at the (P*, $P_A^+P_B^-$) cross peak exhibited similar feature as that at 77 K (Supplementary Figure 7): short-lived high-amplitude oscillation followed by long-lived lower-amplitude oscillation. The short-lived high-amplitude ones reflect electronic coherence [2], so formation of the initial CT state, $P_A^+P_B^-$, from P* is a coherent charge separation process, independent on the temperature. However, it is notable that the oscillation periods were different for the two cases. It was ~190 fs/193 cm^{-1} for 77 K while ~285 fs/129 cm^{-1} for room temperature. We speculated that the difference arose from the temperature dependence of the energy level of $P_A^+P_B^-$.

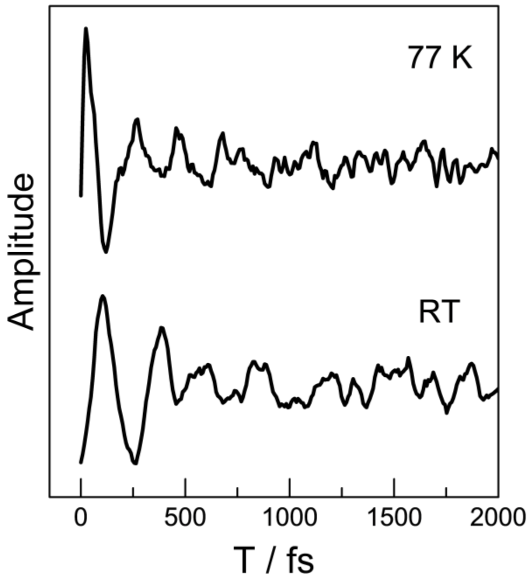

Supplementary Figure 7. QBs at the (P*, $P_A^+P_B^-$) cross peak of **M1** at 77 K (top) and room temperature (bottom). They were real-part T traces at the cross-peak locations labelled as cross in Fig. 3 (77 K) and Supplementary Figure 4 (room temperature) after subtraction of multiexponential dynamics.

Supplementary Note 6

2D frequency maps show characteristic patterns that can help to distinguish electronic, vibrational or mixed coherences [2-5]. The $+\omega_T$ and $-\omega_T$ rephasing frequency maps of the three mutant RCs are shown in Supplementary Figure 8.

For **M1**, they were dominated by diagonal peaks, indicating that they originated from vibrational coherences. In the 115 and 162 cm^{-1} cases, the amplitude of the $-\omega_T$ map is much smaller than the $+\omega_T$ one. Detailed analysis has implied that ground-state vibrational coherences appear exclusively in $\omega_T < 0$ rephasing maps while positive maps feature only excited-state coherences [6]. Thus, the 115 and 162 cm^{-1} oscillations could be assigned as basically excited-state vibrational coherences. It is consistent with the previous conclusion we obtained from the room-temperature 2DES measurement of **M1** [7] that the 153 cm^{-1} oscillation reflected excited-state vibrational coherences. The +35 and −35 cm^{-1} maps had equal amplitudes, thus, it could be assigned to vibrational coherences on both ground and excited states.

It is notable that there was an explicit below-diagonal cross peak in the –115 cm^{-1} frequency map around (860, 910) nm. It was near the (P*, $P_A^+P_B^-$) cross-peak location determined via global analysis, (880, 910) nm (the cross in Supplementary Figure 8). The 20-nm shift along the λ_τ coordinate may reflect the involvement of higher exciton state of P. The appearance of this cross peak indicated that the excited-state vibrational mode with 115 cm^{-1} frequency was associated with the formation of $P_A^+P_B^-$.

For **M2**, the 151 cm^{-1} frequency maps consisted of a main component of diagonal P

peak, a minor component of diagonal B peak and a cross peak around (B, P) position. The 30 cm^{-1} frequency maps contained the same components, but the proportion of B was much higher. The B-related vibrational modes may originate from direct excitation of B or from the backward reaction of electron transfer from P* to B_A. They were assigned to vibrational coherences, however, due to the complexity, it is difficult to distinguish between ground and excited states.

For **M3**, the –115 and –151 cm^{-1} frequency maps were dominated by diagonal P peak, while the +115 and +151 cm^{-1} ones consisted of diagonal P and a cross peak around (B, P) position. They were also assigned to vibrational coherences.

The vibrational coherences of B, although cannot be distinguished between ground and excited states, are more possibly on the ground state. Because they appeared in **M2** and **M3** where the primary ET rates are slower (260 and 25 ps, respectively). During the period of population time (0–2 ps) used to calculate these frequency maps, most B remained on the ground state.

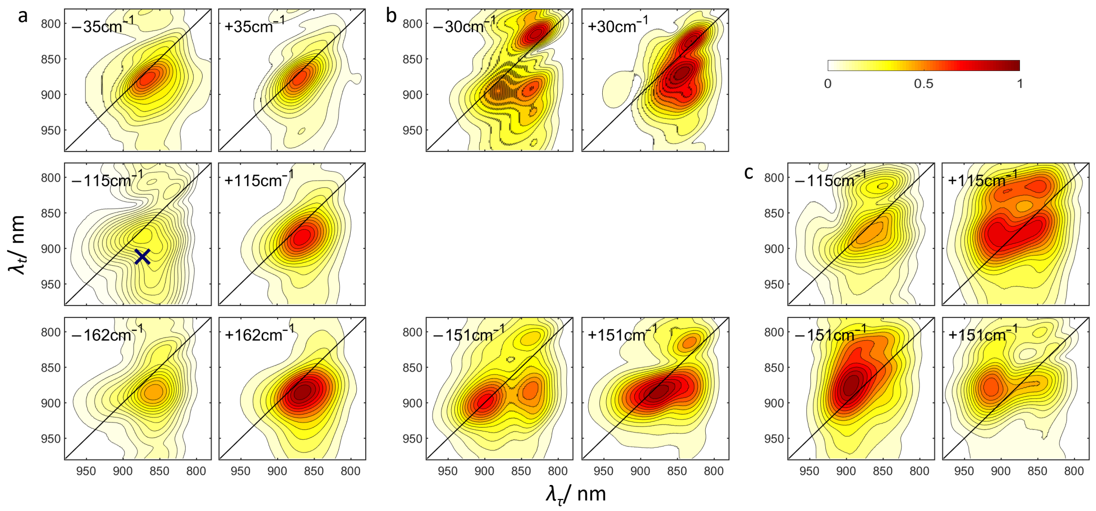

Supplementary Figure 8. 2D frequency maps of M1 (a) M2 (b) and M3 (c) obtained by Fourier transformation of the complex-valued rephasing spectra (ω_T = 0−2ps) after subtraction of the multi-exponential population dynamics. The ω_T frequencies are shown in the left-top corner of each panel. The spectra are normalized to the amplitude of each +162 (or 151) cm^{-1} spectrum. The cross in the −115 cm^{-1} spectrum marks the (P*, $P_A^+P_B^-$) cross-peak location as shown in Fig. 3.

Supplementary References

1 Ma, F., Yu, L. J., Hendrikx, R., Wang-Otomo, Z. Y. & van Grondelle, R. Direct observation of energy detrapping in LH1-RC complex by two-dimensional electronic spectroscopy. *J. Am. Chem. Soc.* **139**, 591−594 (2017).

2 Thyrhaug, E. *et al.* Identification and characterization of diverse coherences in the Fenna-Matthews-Olson complex. *Nat. Chem.* **10**, 780−786 (2018).

3 Butkus, V., Zigmantas, D., Valkunas, L. & Abramavicius, D. Vibrational vs. electronic coherences in 2D spectrum of molecular systems. *Chem. Phys. Lett.* **545**, 40−43 (2012).

4 Romero, E. *et al.* Quantum coherence in photosynthesis for efficient solar-energy conversion. *Nat. Phys.* **10**, 676−682 (2014).

5 de A. Camargo, F. V., Grimmelsmann, L., Anderson, H. L., Meech, S. R. & Heisler, I. A. Resolving vibrational from electronic coherences in two-dimensional electronic spectroscopy: the role of the laser spectrum. *Phys. Rev. Lett.* **118**, 033001 (2017).

6 Butkus, V. *et al.* Discrimination of diverse coherences allows identification of electronic transitions of a molecular nanoring. *J. Phys. Chem. Lett.* **8**, 2344−2349 (2017).

7 Ma, F., Romero, E., Jones, M. R., Novoderezhkin, V. I. & van Grondelle, R. Vibronic coherence in the charge separation process of the *Rhodobacter sphaeroides* reaction center. *J. Phys. Chem. Lett.* **9**, 1827−1832 (2018).
